# NTF2 Upregulation in HNSCC: a Predictive Marker and Potential Therapeutic Target Associated With Immune Infiltration

**DOI:** 10.3389/fonc.2022.783919

**Published:** 2022-06-17

**Authors:** Guangxu Xuan, Xin Zhang, Min Zhang, Minghang Yu, Yujie Zhou, Xiaosong He, Xiaopeng Hu, Xi Wang, Liangfa Liu

**Affiliations:** ^1^Department of Otolaryngology Head and Neck Surgery, Beijing Friendship Hospital, Capital Medical University, Beijing, China; ^2^Department of Otolaryngology Head and Neck Surgery, Affiliated Hospital of Guilin Medical University, Guilin, China; ^3^Department of Urology, Beijing Chao-Yang Hospital, Capital Medical University, Beijing, China; ^4^Beijing Key Laboratory of Emerging Infectious Diseases, Institute of Infectious Diseases, Beijing Ditan Hospital, Capital Medical University, Beijing, China; ^5^Department of Immunology, School of Basic Medical Sciences, Advanced Innovation Center for Human Brain Protection, Beijing Key Laboratory for Cancer Invasion and Metastasis, Department of Oncology, Capital Medical University, Beijing, China; ^6^Beijing Institute of Infectious Diseases, Beijing, China

**Keywords:** NTF2, HNSCC, prognostic biomarker, immune infiltration, immune checkpoint

## Abstract

**Background:**

Head and neck squamous cell carcinoma (HNSCC) is a type of malignant tumor with an increasing incidence worldwide and a meager 5-year survival rate. It is known that nuclear transporter factor 2 (NTF2) transports related proteins into the nucleus physiologically. However, the role of NTF2 in HNSCC remains unclear.

**Methods:**

In this study, RNA-Seq data of HNSCC samples with corresponding clinical information were obtained from The Cancer Genome Atlas (TCGA) database. In addition, other expression profiling data were downloaded from the Gene Expression Omnibus (GEO) database. The differential expressions of NTF2, along with the overall survival (OS) rates were identified and analyzed. Then, the clinical features and expression levels of NTF2 were utilized to develop a prognostic model. The study also utilized the Gene Ontology (GO) and Kyoto Encyclopedia of Genes and Genomes (KEGG) methods to determine the related pathways of NTF2. Furthermore, the Tumor Immune Estimation Resource (TIMER) database was referenced to discover the immune correlation of NTF2. In this research investigation, RT-qPCR, western blotting, Cell Counting Kit-8 (CCK-8) assay, wound-healing assay, and immunohistochemical (IHC) staining methods were adopted to perform experimental verifications.

**Results:**

This study’s results confirmed that the NTF2 expressions were significantly increased in HNSCC tissue when compared with normal tissue. In addition, the high expression levels of NTF2 were found to be associated with poor prognoses, which was confirmed *via* the IHC validations of HNSCC samples with survival data. The results of functional enrichment analysis showed that the NTF2 was associated with epithelial cell growth, skin differentiation, keratosis, and estrogen metabolism. Furthermore, the expressions of NTF2 were determined to be negatively involved with immune infiltrations and correlated with immune checkpoint blockade (ICB) responses following various ICB therapy strategies. The results of the CCK-8 assay and wound-healing assay confirmed the NTF2’s promoting effects on the proliferation and migration of tumor cells.

**Conclusions:**

This study defined a novel prognostic model associated with the expressions of NTF2, which was shown to be independently related to the OS of HNSCC. It was concluded in this study that NTF2 might be a potential diagnostic and prognostic biomarker for HNSCC.

## Introduction

Head and neck squamous cell carcinoma (HNSCC) originates from the mucosal epithelium of the mouth, nasopharynx, oropharynx, hypopharynx, and larynx (1). It is currently the most common malignancy of the head and neck, and the sixth most common cancer globally (2). There are more than 650,000 new cases and 350,000 deaths from HNSCC each year worldwide (3, 4). At present, the incidence of HNSCC is increasing year by year and is expected to increase by 30% by 2030 (5, 6). Tobacco, alcohol, human papillomavirus (HPV), and Epstein-Barr virus (EBV) infections are considered to be risk factors for the high incidence of HNSCC (7–10). It has been determined that due to the asymptomatic nature of the early disease stages, along with the lack of effective screening methods, the majority of patients tend to be diagnosed with advanced squamous cell carcinoma of the head and neck, resulting in a meager 5-year survival rate (11, 12). Therefore, there is an urgent need for effective biomarkers to be identified to assist clinicians in accurately predicting clinical outcomes and provide references for personalized medical treatments to combat HNSCC.

It has long been noted that the size of the nucleus tends to correlate with the size of the cell (13–17). Nuclear transport factor 2 (NTF2, also known as NUTF2) is bound up with nuclear size regulation and was initially identified based on its ability to stimulate nuclear input in permeable cells (18, 19). It has subsequently been shown to be responsible for importing Ran-GDP into the nucleus (20, 21). It has been found that altered nuclear scaling is associated with many types of cancer, and pathologists monitor the increased grading of nuclear sizes in cancer diagnosis and prognosis processes (22, 23). It has been reported that increased nuclear size during melanoma progression is related to decreased NTF2 expressions. In addition, increased NTF2 levels in melanoma cells are known to be sufficient for reducing the nuclear size (24). While Du et al. reported that NTF2 overexpression promoted the proliferation, migration, and invasion of glioma cells, which suggests that NTF2 is an oncogene in glioma (25). However, at present, the function of NTF2 in HNSCC remains unclear.

In this study, RNA sequencing data and the corresponding clinical information of HSNCC patients from the Cancer Genome Atlas (TCGA) database were comprehensively analyzed. The differential expressions of NTF2 were examined, and its diagnostic and prognostic values were evaluated. The results were further validated with clinical patients. The relationships between the expression levels of NTF2 and immune infiltration were then analyzed, and the function of NTF2 in HNSCC cell lines was verified. Finally, NTF2 was successfully identified as a potential diagnostic and prognostic biomarker for HNSCC.

## Materials and Methods

### Downloaded Data and Differential Expression Analysis

In this research investigation, the original counts and corresponding clinical information of RNA sequencing data (Level 3) for 528 HNSCC samples and 44 normal samples were obtained on July 1^st^, 2021 from TCGA dataset (https://portal.gdc.cancer.gov/). Also, the expression profiling data of 22 pairs of HNSCC were obtained from the Gene Expression Omnibus (GEO) database (https://www.ncbi.nlm.nih.gov/geo/). The data were analyzed in SPSS (Version 25, IBM Corp., USA), and the results were processed using Graphpad Prism (Version 8, GraphPad Software, USA). In addition, the UALCAN database (http://ualcan.path.uab.edu/) was referenced to investigate the relationships between the expression levels of NTF2 and various clinical features.

### Survival Analysis

In this study, the prognostic values were determined by the Kaplan-Meier curves, and univariate and multivariate Cox regression analysis was performed. The R packages “RMS” and “RMDA” packages were utilized to perform the nomogram, calibration, and decision curve analysis (DCA) based on the results of the multivariate Cox proportional risk analysis. Nomogram and DCA were used to evaluate and compare the predictive models containing the clinical outcomes. All of the above-mentioned analysis methods and R packages were performed using R software version 4.0.3, and P < 0.05 was considered to be statistically significant.

### Functional Enrichment Analysis

Differentially expressed genes were identified and analyzed using the “limma” R package and wilcoxon tests based on the expression levels of the NTF2 (26, 27). A false discovery rate (FDR) < 0.05 and a |log2(fold change)| >1 were set as the thresholds. Then, Gene Ontology (GO) and the Kyoto Encyclopedia of Genes and Genomes (KEGG) were utilized when using the “cluster profiler” R package. Finally, the “ggplot2” R package was adopted to visualize the experimental results.

### Correlation Analysis of the NTF2 *via* Immune Infiltration, Immune Checkpoints, and ICB Responses

The Tumor Immune Estimation Resource (TIMER) database (https://cistrome.shinyapps.io/timer/) was referenced in this study to analyze six subsets of tumor-infiltrating immune cells (28). The immune checkpoint-related genes (SIGLEC15, TIGIT, CD274, HAVCR2, PDCD1, CTLA4, LAG3, and PDCD1LG2) were extracted from TCGA database to explore the tumor immunology. Then, Tumor Immune Dysfunction and Exclusion (TIDE) was used to predict the potential immune checkpoint blocking (ICB) responses (29). The R software packages “ggplot2”, “pheatmap”, and “ggpubr” were used in this research for graph visualization. In the aforementioned analysis processes, a P value of less than 0.05 was considered to be statistically significant.

### Cell Cultures

During this study’s experimental processes, human nasopharyngeal carcinoma cell line 5-8F cells and human pharyngeal squamous carcinoma cell line Fadu cells were cultured in RPMI1640 (iCell, China) and MEM medium (Gibco, USA), respectively. All of the media contained 10% heat-inactivated fetal bovine serum (FBS) and 1% penicillin/streptomycin. Also, all of the cells were incubated at 37°C under the condition of 5% CO_2_.

### siRNA Transfection, RNA Isolation, and Real-Time qPCR Methods

The NTF2 was knocked down in 5-8F and Fadu cell lines via siRNA transfection (Hanbio, China). Then, the 5-8F and Fadu cells were collected for subsequent studies after transfection for 48 hours.

The total RNA was extracted according to the instructions of the Trizol Reagent (Invitrogen, USA), and real-time quantitative PCR (RT-qPCR) was performed using SYBR Green (Vazyme, China). The relative expression levels of the genes were analyzed using the ΔΔCT method and normalized to GAPDH.

The specific primers and siRNA sequences were listed in **Table 1**.

**Table 1:** Primer and siRNA sequences of related genes.

Gene		Sequence (5’-3’)
Primer-NTF2	F*	AACCCAACTAGGCGCAATTTA
	R	ACGGAAGGCTAGACAACTTCT
Primer-GAPDH	F	AAGAAGGTGGTGAAGCAGGC
	R	GAGTGGGTGTCGCTGTTGAA
siRNA-NTF2		
si1	F	CCACCAGAUGUUCCUAUUAAATT
	R	UUUAAUAGGAACAUCUGGUGGTT
si2	F	GAUGCUUGGGUUUGCACCAAUTT
	R	AUUGGUGCAAACCCAAGCAUCTT
siRNA-GAPDH	F	UAAAGUACCCUGUGCUCAATT
	R	UUGAGCACAGGGUACUUUATT

*F, forward; R, reverse.

### Western Blotting

In order to assess the protein expression levels of the NTF2, a RIPA buffer and protease inhibitors (Gene-Protein Link, China) were used to disrupt the cells. Then, 20 μg of total protein was separated on 15% SDS-PAGE gel. The antibodies specific for NTF2 (66063-1, Proteintech, China) (1:1000) and GAPDH (T004, Affinity, China) (1:2000) were utilized for probing the proteins. Then, specific proteins were visualized using the method provided by the AI600 Imaging System (GE, USA).

### Wound-Healing Assay

In the present study, 5-8F cells were inoculated in 6-well plates containing MEM with 10% FBS at a density of 4 × 10^5^ cells/well. Subsequently, when 90% of the cells had become fused after 16 to 24 hours of culture, the confluence cells were lined with a 200 µL pipette. Then, the injured monolayer cells were washed with phosphate-buffered saline (PBS) for the purpose of removing the cell debris. In the next experimental step, the MEM with 10% FBS was replaced with a serum-free medium. Images were obtained at 0, 6, and 24 hours, and each experiment was independently performed at least three times. The scratch areas were evaluated using Image J, and the cell migration rates were calculated using the following formula:


Migration rate (%)=(original area−measured area)/original area×100%

### Immunohistochemical Staining

Paraffin-embedded tissue was selected from 66 HNSCC patients who had undergone surgery in the Affiliated Hospital of Guilin Medical University between April 2012 and October 2019. The patients enrolled in this study were diagnosed with primary squamous cell carcinoma of the larynx, with no other malignancies in the mouth, oropharynx, or pharynx, and no previous history of radiotherapy or chemotherapy. In addition, both clinical and pathological data were collected, including the patients’ ages, differentiation grades, lymph node metastasis, and survival periods. The follow-up and postoperative management data of the patients were collected by telephone or from outpatient medical records. The tumor stages were classified according to the tumor node metastasis (TNM) staging system (2017) of the Union for International Cancer Control (UICC). The samples obtained from the 66 HNSCC patients and ten adjacent normal tissue were each divided into 4 µm thick sections for this study’s immunohistochemical (IHC) analysis process. A primary monoclonal antibody (66063-1, Proteintech, China) was used to detect the expression levels of the NTF2. Secondary antibodies to mouse IgG were obtained from the IHC kit (#CW2069, Beijing Cowin Bioscience Co., Ltd., China). Mouse IgG was used as a negative control to exclude false positive results. The staining intensity was assessed by histology score (H-score) and semi-quantitative analysis was performed by two independent pathologists who had not been informed of the sources of the clinical samples (30, 31). The following formula was applied:


H−score=(percentage of weak intensity cells ×1)+(percentage of moderate intensity cells ×2)+(percentage of strong intensity cells ×3)

The studies involving human participants were reviewed and approved by the Ethics Committee of the Affiliated Hospital of Guilin Medical University. All of the patients/participants provided their written informed consent to participate in this study.

### Statistical Analysis

Two-tailed student t-tests were performed in SPSS to evaluate the statistical significance in this study. The significance differences of P < 0.05, 0.01, and 0.001 were symbolized as *, **, and ***, respectively.

## Results

### NTF2 Was Highly Expressed in HNSCC

**Figure 1:**
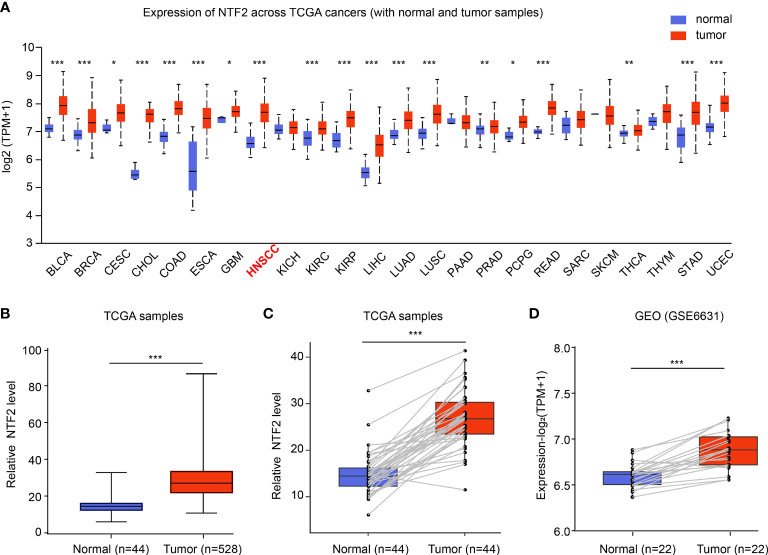
Expression of NTF2 in HNSCC. **(A)** NTF2 expressions in different types of cancers were examined using the UALCAN database. **(B)** Analysis of NTF2 expression in HNSCC using TCGA database. Comparison of NTF2 mRNA levels in paired adjacent normal tissue and tumor tissue of HNSCC from TCGA **(C)** and GEO database **(D)**. *P < 0.05, **P < 0.01, ***P < 0.001.

The mRNA expressions of NTF2 in human cancer cells were analyzed using the UALCAN database. It was found that when compared with the corresponding normal tissue, higher expressions of NTF2 were observed in the majority of the cancer types, including HNSCC (P < 0.001); bladder urothelial carcinoma (BLCA); breast invasive carcinoma (BRCA); cholangiocarcinoma (CHOL); colon adenocarcinoma (COAD); esophageal carcinoma (ESCA), and so on (**Figures 1A, B**). In addition, significant increases in the NTF2 expressions in HNSCC cases were observed in 44 cases of tumor tissue with paired adjacent normal tissue (P < 0.001) (**Figure 1C**). Furthermore, similar results were observed in 22 pairs of HNSCC samples from the GSE6631 cohort in the GEO database (P < 0.001) (**Figure 1D**). Therefore, the findings suggested that NTF2 may play a vital regulatory role in the development and progression of HNSCC.

### NTF2 Expression Levels and the Clinical Features of the HNSCC Patients

**Figure 2:**
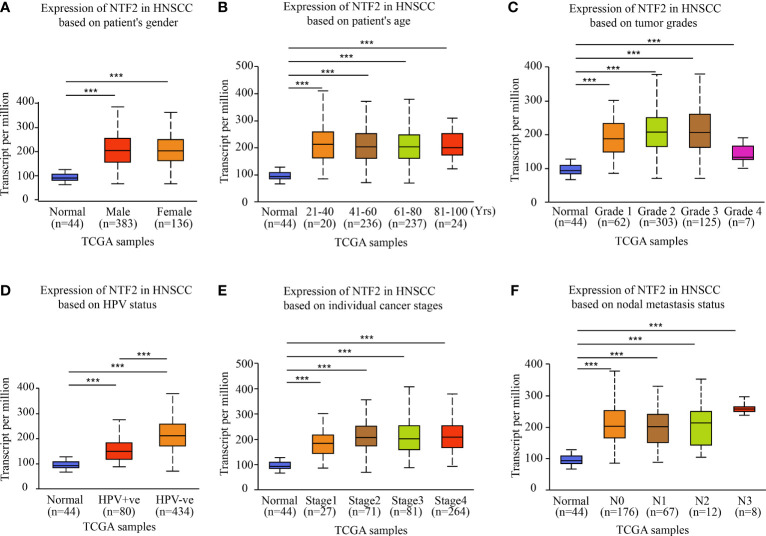
NTF2 expressions in different groups were evaluated according to clinical features based on UALCAN database. Analysis was shown for sex **(A)**, age **(B)**, pathological grade **(C)**, HPV infection **(D)**, clinical-stage **(E)**, and lymph node metastasis status **(F)**. N0: no regional lymph node metastasis; N1: 1 to 3 cervical lymph nodes metastasis; N2: 4 to 9 cervical lymph nodes metastasis; N3: 10 or more cervical lymph nodes metastasis. ***P < 0.001.

This study investigated the NTF2 expression levels in various HNSCC subgroups using the UALCAN database. It was observed that the NTF2 expression levels were higher in the HPV negative group than in the positive group (P < 0.001; **Figure 2D**). In addition, the NTF2 expression levels were significantly up-regulated in both the men and women tumor groups, respectively (P < 0.001) (**Figure 2A**). The same results were observed for the different age groups (P < 0.001; **Figure 2B**); pathological grade groups (P < 0.001; **Figure 2C**); HPV infection groups (P < 0.001; **Figure 2D**); tumor stage groups (P < 0.001; **Figure 2E**); and lymph node metastasis groups (P < 0.001; **Figure 2F**) among the HNSCC cases. However, there were no significant differences observed among the clinical subgroups.

### High Expressions of NTF2 Were Observed to be Correlated With the Poor Prognoses of the HNSCC Patients

**Figure 3:**
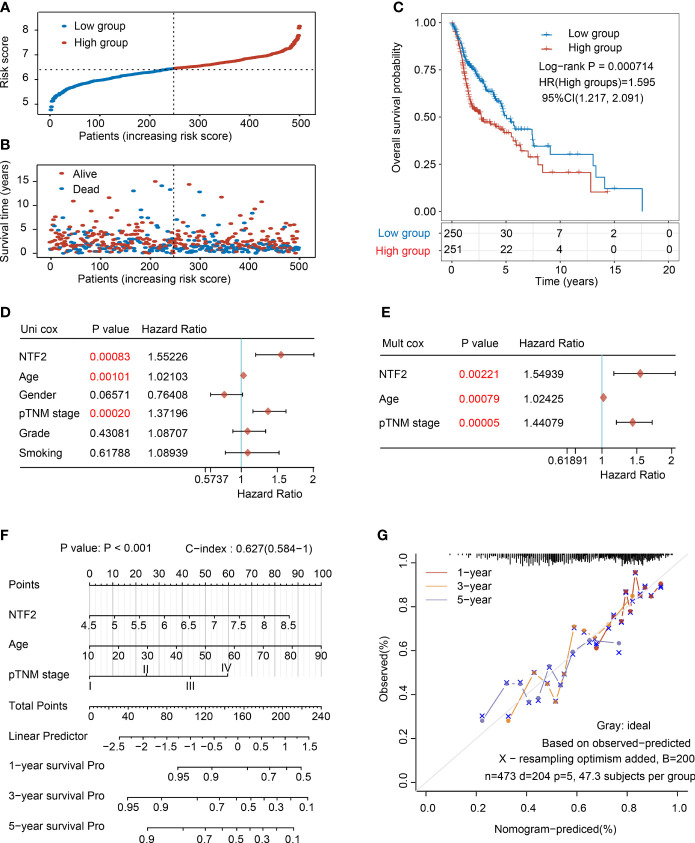
Analysis of the prognostic risk signature based on NTF2 expression in TCGA database. **(A)** The risk score distribution of HNSCC patients. **(B)** Survival status and duration of patients. **(C)** Survival curve of NTF2 with high and low expression. The univariate **(D)** and multivariate **(E)** independent prognostic analysis of independent risk factors for overall survival (OS) in HNSCC patients. **(F)** Nomogram to predict the 1-, 3-, and 5-year overall survival of HNSCC patients. **(G)** Calibration curve for the OS nomogram model. The grey dotted line represents the ideal prediction curve.

Then, the prognostic values of the NTF2 were determined. Following a median expression level, the patients were divided into the following two groups: High expression group (n = 264) and low-expression group (n = 264), as detailed in **Figure 3A**. The patients in the high expression group were observed to have remarkably higher mortality rates than those in the low-expression group (**Figure 3B**). In addition, the Kaplan-Meier survival curves also showed that the survival rates of the high expression patients were significantly lower than those of the low-expression patients (P = 0.000714; **Figure 3C**).

In the present investigation, both univariate and multivariate Cox proportional risk analyses were performed. The results revealed that the NTF2 expression levels, patient ages, and TNM stages were independent prognostic factors (**Figures 3D, E**), which were included to establish an accurate prediction model. This study’s nomogram provided a graphical representation of the aforementioned factors, and the prognostic risks for an individual patient could be calculated by the points associated with each risk factor, as detailed in **Figure 3F**. In addition, as shown in **Figure 3G**, the calibration plots showed excellent agreement between the actual probabilities and the estimated probabilities at 1, 3, and 5 years.

### Experimental Verifications of the Clinical Samples

**Figure 4:**
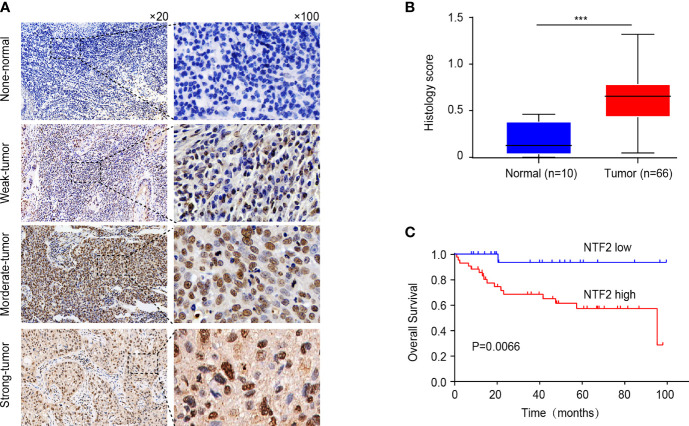
Immunohistochemical evaluation of NTF2 as a prognostic marker. **(A)** Negative, weak, moderate, strong immunohistochemical staining of NTF2 was shown respectively in HNSCC and normal samples. **(B)** The H-scores of 66 HNSCC tissue were compared with those of 10 normal tissue. **(C)** Overall survival rate was compared between low and high expression group of NTF2 based on H-score. ***P < 0.001.

An immunohistochemical staining method was used to detect the expression levels of NTF2 in the tumor samples from 66 HNSCC patients and 10 normal tissue samples. The results revealed that the NTF2 was highly expressed in the HNSCC tissue when compared with the normal tissue (**Figures 4A, B**). Meanwhile, there was no significant correlation observed between the NTF2 expression levels and the patient ages, genders, pathological grades, tumor stages, lymph node metastasis, or smoking habits (**Table 2**). The median follow-up timeframe for all of the examined patient cases was 36.2 months (ranging from 1.0 to 99.9 months). At the final follow-up times, it was determined that in 49 cases (74.2%), the patients had survived, and in 17 cases (25.8%) the patients had died. The Kaplan-Meier analysis results showed that the high expression levels of NTF2 were closely related to significant reductions in overall survival (P = 0.0066) in the HNSCC case samples (**Figure 4C**).

**Table 2:** Correlation between NTF2 expression and the clinicopathological features in 66 HNSCC samples.

Characteristics	NTF2 expression		P value
	High (n = 42)	Low (n = 24)	
**Age (years, mean ± SD)**	62.36 ± 9.414	61.46 ± 5.956	0.675
**Sex (M/F)**	42/0	24/1	0.1825
**Pathological differentiation**			
Well	30	14	
Moderate/poor	12	10	0.2776
**TNM Stage**			
T1,T2	17	15	
T3,T4	25	9	0.085
**Lymph node metastasis**			
Yes	12	5	
No	30	19	0.4892
**Smoking**			
Yes	32	18	
No	10	6	0.9135

### Functional Enrichment Analysis

**Figure 5:**
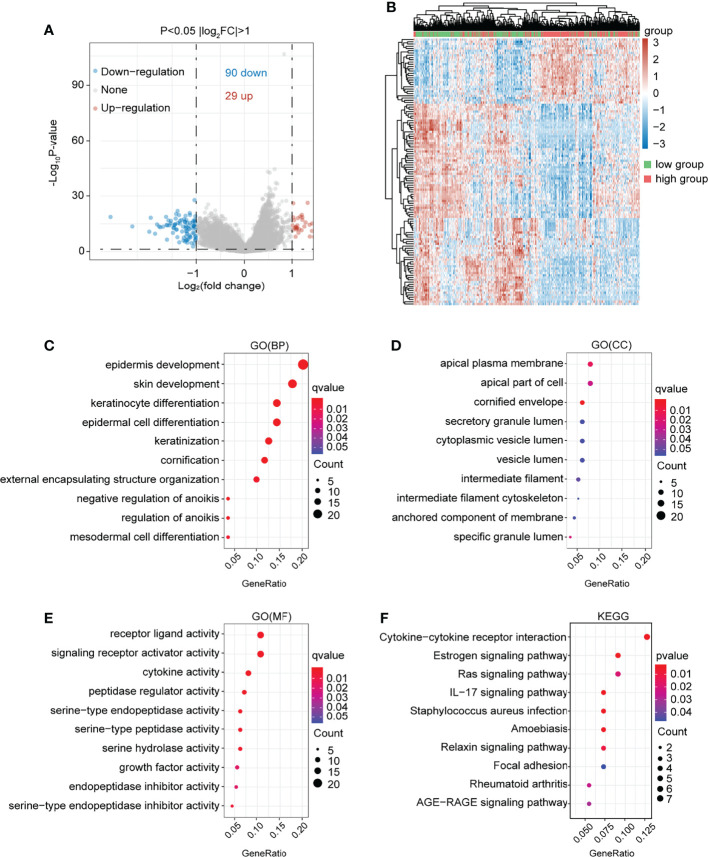
Functional enrichment analysis of NTF2 related genes. **(A)** In the volcano map of HNSCC in TCGA database, red dots were up-regulated genes and blue dots were down-regulated genes. **(B)** Heatmap of differential genes between NTF2 high and low groups in TCGA database. **(C–E)** GO analyses. **(F)** KEGG analyses.

In the present study, the co-expressed genes related to NTF2 were identified by mining data from TCGA database. This study’s volcano map and heat map with positive and negative correlations with NTF2 in HNSCC were shown in **Figures 5A, B**, respectively. A total of 119 genes associated with NTF2 (P < 0.05) were used in the GO and KEGG enrichment analyses in order to explore relevant biological functions and pathways. The top 30 critical terms for the enrichment analysis of the biological processes (BP), cellular components (CC), and molecular functions (MF) were detailed in **Figures 5C–E**. The first 10 KEGG pathways of the related genes were shown in **Figure 5F**.

### Correlation Analysis of NTF2 Expression Level With Immune Infiltration, Immune Checkpoint, and ICB Response

**Figure 6:**
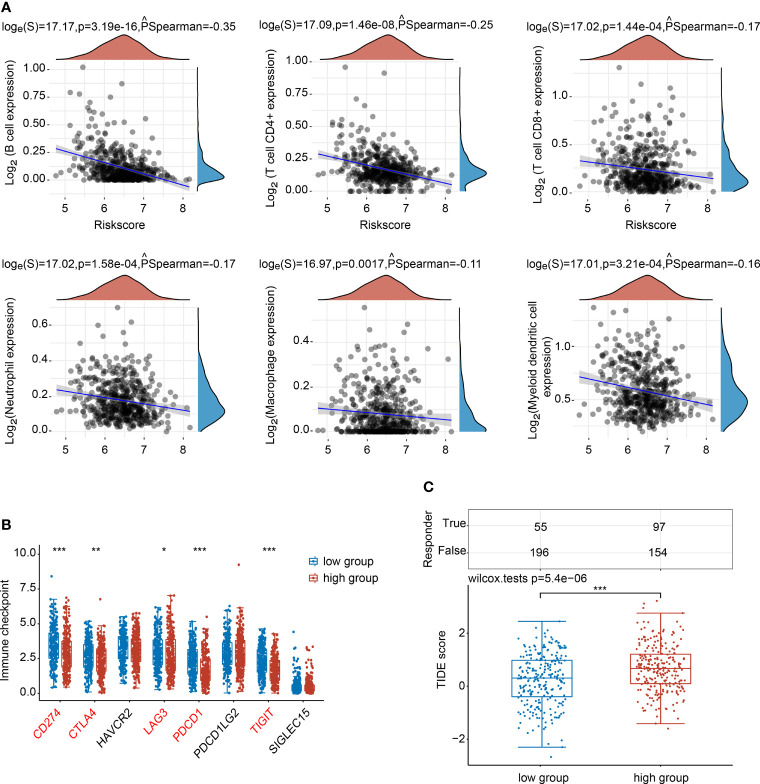
Correlations between NTF2 and immune status in HNSCC patients. **(A)** Spearman analysis between NTF2 and immune score. **(B)** Correlation analysis between NTF2 and immune checkpoint-related gene expression using Wilcox on test. **(C)** Potential immunotherapeutic responses were predicted through the TIDE algorithm. *P < 0.05, **P < 0.01, ***P < 0.001.

The associations between the NTF2 expression levels and the infiltrating immune cells were analyzed. The results showed that NTF2 expression levels were negatively correlated with the B cells (PSpearman = -0.35; P < 0.001); CD4^+^ T cells (PSpearman = -0.25; P < 0.001); CD8^+^ T cells (PSpearman = -0.17; P < 0.001); neutrophils (PSpearman = -0.17; P < 0.001); macrophages (PSpearman = -0.11; P = 0.0017); and dendritic cells (PSpearman = -0.16; P < 0.001) (**Figure 6A**). The immune checkpoint-related genes (SIGLEC15, TIGIT, CD274, HAVCR2, PDCD1, CTLA4, LAG3, and PDCD1LG2) were extracted and analyzed. It was found that the CD274, CTLA4, LAG3, PDCD1, and TIGIT genes displayed negative correlations with the NTF2 expressions (**Figure 6B**). The potential ICB responses indicated that the NTF2 high expression group had a poor efficacy for immune checkpoint blockade treatments (**Figure 6C**).

### NTF2 Regulation of the Proliferation and Migration of HNSCC Cells

**Figure 7:**
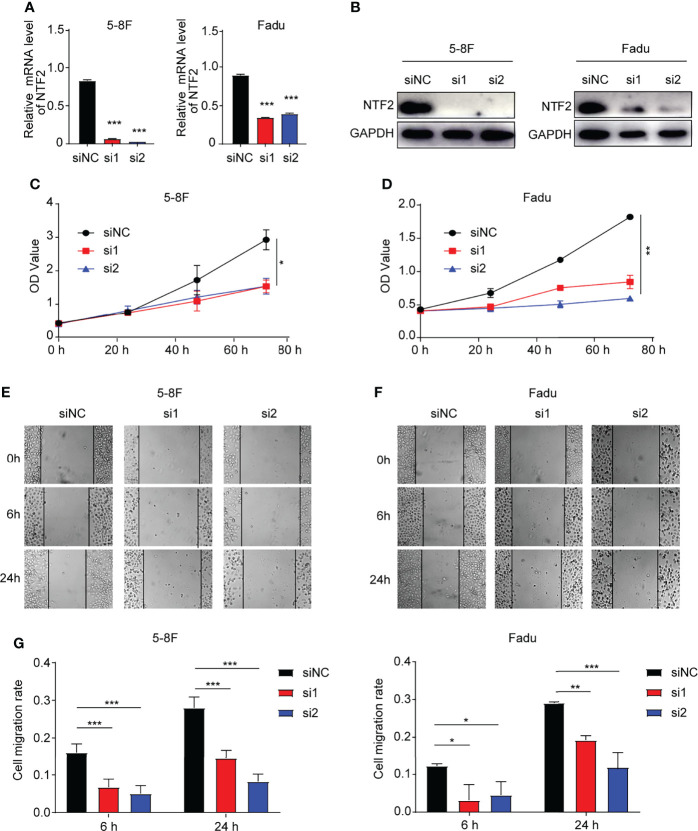
Experimental validation. **(A, B)** Knockdown validation by qRT-qPCR and western blotting. CCK-8 assay **(C, D)** and wound-healing assay **(E, F)** were used to detect the growth and migration of NTF2-knockdown HNSCC cell lines. *P < 0.05, **P < 0.01, ***P < 0.001.

In order to investigate the role of NTF2 in HNSCC cells, two siRNAs targeting NTF2 (si1, si2) were transfected into 5-8F and Fadu cells respectively. It was found that when compared with the control cells treated with empty vector, the NTF2 was significantly silenced at the mRNA (**Figure 7A**) and protein levels in the knockdown group (**Figure 7B**). This study then tested the effects of cell proliferation *in-vitro*. Using the CCK-8 assay, it was found that the knockdown of the NTF2 could inhibit HNSCC cell proliferation after 48 hours (P < 0.001) and 72 hours (P < 0.001) of culturing (**Figures 7C, D**). In addition, the wound-healing assay was used to assess the capacity of cancer cell migration. The results revealed that the cells treated with NTF2 siRNAs showed lower migration rates than the control cells after 6 hours (P < 0.001) and 24 hours (P < 0.001) of culturing (**Figures 7E, F**).

## Discussion

Following the Global Burden of Disease study, the incidence of lip and oral cancers has increased by 36.5%, throat cancers by 23.1%, and other pharyngeal cancers by 29.9% over the past decade (32, 33). It has been found that with the increased stages of the tumor, the survival rates of HNSCC patients decreased, and the postoperative recurrence rate increased (34–36), which could not be improved by adjustments in treatment regimens (37, 38). Therefore, the development of new therapeutic targets and prognostic markers is urgently required. In previous investigations, NTF2 had been reported to reduce the nuclear sizes of melanoma cells and was found to be highly expressed in glioma tissue (24, 25). However, NTF2 had not yet been reported in HNSCC cases. This study found increased expression levels of NTF2 in TCGA and GEO databases, which was confirmed by the results obtained in this study’s tissue samples. In addition, knockdown verifications of this molecule were conducted for the first time in the current investigation. The results confirmed that the downregulation of NTF2 could inhibit HNSCC cells proliferation and migration.

HPV infections are known to be associated with the majority of oropharyngeal cancers (> 70%) and are considered to be increasingly common risk factors for HNSCC (39, 40). HPV-associated tumors are modulated by helical domain mutations of the oncogene PIK3CA, loss of TRAF3, and the amplification of the cell cycle gene E2F1 (41). In this study, there was observed to be significant statistical differences in the expression levels of NTF2 between the HPV infection group and the non-HPV infection group. Therefore, the results suggested that the NTF2 may be involved in the integration of the HPV’s genetic information into the host genome.

Although such clinical indicators as TNM can be used to judge the prognoses of patients, they still have certain limitations (42). At present, the accumulation of public genome databases and the recent advances in bioinformatics have made it possible to acquire a comprehensive cancer genome map in large cohorts (43). However, the effects of NTF2 on tumor survival in HNSCC remain under-reported. The results obtained in this study showed that the high expressions of NUFT2 were related to the poor prognostic outcomes of the HNSCC patients in the bioinformatics database. Therefore, a nomogram was constructed in this study for the comprehensive predictions of patient survival rates in clinical settings. In addition, the prognostic effects were reconfirmed by the collected tissue samples.

TIMER web server is a comprehensive resource for the systematic analysis of immune infiltrates across diverse cancer types (28, 44). The relationships between the NTF2 expression levels and the tumor-infiltrating immune cells were analyzed in this study using the TIMER database. It was found that the NTF2 expression levels were negatively correlated with six immune cells. Therefore, it was indicated that NTF2 might indirectly alter tumor immune microenvironments. Furthermore, this study considered that immune checkpoint therapy may be less effective in patients with high expressions of NTF2, suggesting that it was a predictor of malignant prognosis.

However, it should be noted that there were still some limitations in this study. For example, the HPV infection data were not available in the clinical data. In addition, although the functions of NTF2 in cells were initially explored, the mechanisms of those functions were not investigated. Therefore, further studies should be conducted *in-vivo* and *in-vitro* to investigate the functions and mechanisms of NTF2 in HNSCC.

In conclusion, the results obtained in this study elucidated the differential expressions and clinical prognosis values of NTF2. The NTF2 immune-related functions were also discussed, which reflected the clinical and biological significance of NTF2 in HNSCC. The obtained results suggested that the NTF2 might be a potential novel tumor prognostic marker and therapeutic target in the future.

## Data Availability Statement

The datasets presented in this study can be found in online repositories. The names of the repository/repositories and accession number(s) can be found in the article/supplementary material.

## Ethics Statement

The studies involving human participants were reviewed and approved by The Ethics Committee of Affiliated Hospital of Guilin Medical University. The patients/participants provided their written informed consent to participate in this study.

## Author Contributions

LL, XW, and XHu reviewed the manuscript and supervised the study. GX carried out the experiments, exported the figures, and wrote the first draft of the manuscript. GX, XZ, and MZ designed the research processes and finalized the manuscript. GX, MY, YZ, and XHe collected the clinical samples used in this investigation. All authors contributed to the article and approved the final manuscript.

## Funding

This study was supported by the Research and Development Project of Scientific Research Instruments and Equipment of the Chinese Academy of Sciences - major instruments project (YJKYYQ20180039) and the Digestive Medical Coordinated Development Center of Beijing Municipal Administration of Hospitals (No. XXZ0604), the Support Project of High-level Teachers in Beijing Municipal Universities in the Period of 13th Five-year Plan (IDHT20190510 to XW), and the National Natural Science Foundation of China (grant# 81972652 & 81171899 to XW).

## Conflict of Interest

The authors declare that the research was conducted in the absence of any commercial or financial relationships that could be construed as a potential conflict of interest.

## Publisher’s Note

All claims expressed in this article are solely those of the authors and do not necessarily represent those of their affiliated organizations, or those of the publisher, the editors and the reviewers. Any product that may be evaluated in this article, or claim that may be made by its manufacturer, is not guaranteed or endorsed by the publisher.
